# Plasma acylcarnitines and gut‐derived aromatic amino acids as sex‐specific hub metabolites of the human aging metabolome

**DOI:** 10.1111/acel.13821

**Published:** 2023-03-23

**Authors:** Joaquim Sol, Èlia Obis, Natalia Mota‐Martorell, Irene Pradas, Jose Daniel Galo‐Licona, Meritxell Martin‐Garí, Anna Fernández‐Bernal, Marta Ortega‐Bravo, Jordi Mayneris‐Perxachs, Consuelo Borrás, José Viña, Mónica de la Fuente, Ianire Mate, Carles Biarnes, Salvador Pedraza, Joan C. Vilanova, Ramon Brugada, Rafel Ramos, Joaquin Serena, Lluís Ramió‐Torrentà, Víctor Pineda, Pepus Daunis‐I‐Estadella, Santiago Thió‐Henestrosa, Jordi Barretina, Josep Garre‐Olmo, Manuel Portero‐Otin, José Manuel Fernández‐Real, Josep Puig, Mariona Jové, Reinald Pamplona

**Affiliations:** ^1^ Department of Experimental Medicine University of Lleida‐Lleida Biomedical Research Institute (UdL‐IRBLleida) Lleida Spain; ^2^ Research Support Unit (USR) Lleida, Primary Care Services Catalan Health Institute (ICS) Lleida Spain; ^3^ Fundació Institut Universitari per a la Recerca en Atenció Primària de Salut Jordi Gol i Gurina (IDIAP JGol) Lleida Spain; ^4^ Girona Biomedical Research Institute (IDIBGI) Hospital Universitari de Girona Dr Josep Trueta Girona Spain; ^5^ Department of Nursing University of Girona Girona Spain; ^6^ Department of Diabetes, Endocrinology and Nutrition, IDIBGI Hospital Universitari de Girona Dr Josep Trueta Girona Spain; ^7^ CIBER Fisiopatología de la Obesidad y Nutrición (CIBERobn) Madrid Spain; ^8^ Freshage Research Group, Department of Physiology, Faculty of Medicine University of Valencia, Centro de Investigación Biomédica en Red Fragilidad y Envejecimiento Saludable‐Instituto de Salud Carlos III (CIBERFES‐ISCIII), INCLIVA Valencia Spain; ^9^ Department of Genetics, Physiology and Microbiology, Facultad de Ciencias Biológicas Universidad Complutense de Madrid Madrid Spain; ^10^ Department of Radiology (IDI) Hospital Universitari de Girona Dr Josep Trueta Girona Spain; ^11^ Cardiovascular Genetics Center, CIBER‐CV, IDIBGI Girona Spain; ^12^ Vascular Health Research Group of Girona (ISV‐Girona) Institut Universitari d'Investigació en Atenció Primària Jordi Gol (IDIAP Jordi Gol) Girona Spain; ^13^ Primary Care Services Catalan Institute of Health (ICS) Girona Spain; ^14^ Department of Neurology Hospital Universitari de Girona Dr Josep Trueta Girona Spain; ^15^ Department of Computer Science, Applied Mathematics and Statistics University of Girona Girona Spain; ^16^ Institut Investigació Germans Trias i Pujol (IGTP) Comparative Medicine and Bioimage of Catalonia Barcelona Spain

**Keywords:** aging, aromatic amino acids, bioenergetics, liquid chromatography‐mass spectrometry, metabolomics, sex/gender perspective

## Abstract

Aging biology entails a cell/tissue deregulated metabolism that affects all levels of biological organization. Therefore, the application of “omic” techniques that are closer to phenotype, such as metabolomics, to the study of the aging process should be a turning point in the definition of cellular processes involved. The main objective of the present study was to describe the changes in plasma metabolome associated with biological aging and the role of sex in the metabolic regulation during aging. A high‐throughput untargeted metabolomic analysis was applied in plasma samples to detect hub metabolites and biomarkers of aging incorporating a sex/gender perspective. A cohort of 1030 healthy human adults (45.9% females, and 54.1% males) from 50 to 98 years of age was used. Results were validated using two independent cohorts (1: *n* = 146, 53% females, 30–100 years old; 2: *n* = 68, 70% females, 19–107 years old). Metabolites related to lipid and aromatic amino acid (AAA) metabolisms arose as the main metabolic pathways affected by age, with a high influence of sex. Globally, we describe changes in bioenergetic pathways that point to a decrease in mitochondrial β‐oxidation and an accumulation of unsaturated fatty acids and acylcarnitines that could be responsible for the increment of oxidative damage and inflammation characteristic of this physiological process. Furthermore, we describe for the first time the importance of gut‐derived AAA catabolites in the aging process describing novel biomarkers that could contribute to better understand this physiological process but also age‐related diseases.

## Graphical abstract

A high‐throughput untargeted metabolomic analysis applied to plasma samples of 1030 healthy human adults from 50 to 98 years of age revealed acylcarnitines and gut‐derived aromatic amino acid catabolites as hub metabolites and biomarkers of aging revealing a high influence of sex. These results were mainly validated using independent cohorts and bring us closer to the description of the phenotype of this universal process being essential to understand the physiopathology of aging.

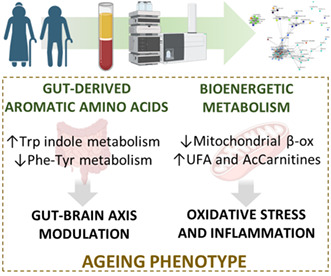

**Abbreviations**
AA: arachidonic acid
AAA: aromatic amino acid
AcCar: acylcarnitine
BM: biomarkers
BNZ: benzene and substituted derivatives
CA: carboxylic acids and derivatives
DG: diacylglyceride
DPA: docosapentaenoic acid
ESI‐Q‐TOF: electrospray ionization quadrupole time of flight
FA: fatty acyls
FAHFA: fatty acyl esters of hydroxy fatty acid
FDR: false discovery rate
GL: glycerolipids
GPL: glycerophospholipids
HA: hydroxy acids and derivatives
HMDB: human metabolome data base
IND: indoles
lysoPC: lysophosphatidylcholine
lysoPE: lysophosphatidylethanolamine
MS/MS: tandem mass spectrometry
MS: mass spectrometry
NA: nervonic acid
OA: oleic acid
OCA: organic carbonic acids and derivatives
ON: organonitrogen compounds
PA: phosphatidic acid
PC: phosphocholine
PCA: principal component analyses
PE: phosphoethanolamine
PG: phosphoglycerol
PHE: phenols
PIP: phosphoinositol phosphate
PL: prenol lipids
PS: phosphoserine
ST: steroids and steroid derivatives
TCA: trycarboxylic acid cycle
TP: tetrapyrroles and derivatives
UHPLC: ultra‐high‐performance liquid chromatography

## 1 INTRODUCTION

Aging is an endogenous and deleterious physiological process that affects all levels of biological organization (from genome to metabolome, and from cell metabolism to organism) leading to a cell/tissue‐specific loss of function (Jové, Portero‐Otín, et al., 2014). Consequently, the ability to maintain homeostasis decreases and requires a continuous and dynamic adaptive response. The changes induced by aging become, depending on the individual vulnerability, the substrate for the appearance of age‐related diseases such as cancer and degenerative processes (Butler et al., 2008) thus increasing the risk of morbidity and mortality. In line with this, age‐related diseases can be interpreted as a manifestation of accelerated aging and have a great impact on healthspan and quality of life. Therefore, it is basic to understand the physiology of aging to provide insights into mechanisms of disease to allow populations to achieve pathology‐free advanced ages with an optimal quality of life.

In recent decades, many efforts have been focused on defining biomarkers (BM) of aging (Johnson, 2006). In this sense, the application of “omic” techniques has a revolutionary role in offering a wide range of potential BM (Valdes et al., 2013) in order to be able to understand and modulate the aging process. For achieving this purpose, blood plasma (and serum) arises as a relevant sample of study, as they are relatively easy to obtain and have important impact in modulating aging. For instance, the infusion of plasma from old to young mice is sufficient to accelerate brain aging (Villeda et al., 2011) and young plasma can partially reverse this physiological process (Villeda et al., 2014). These results suggest that the components of the plasma are key components related to aging and indicate that applying “omic” techniques that are closer to phenotype, such as metabolomics, should be a turning point in the definition of the cellular processes involved.

Plasma is a primary carrier of metabolites in the body (containing more than 18,500 different species [Wishart et al., 2018]), and its metabolome expresses cellular needs and cell‐tissue metabolism. Furthermore, the plasma metabolome profile is species‐specific and is an optimized feature associated with animal longevity (Jové et al., 2013). Therefore, the aging process, as well as age‐related diseases, should have an impact on the plasma metabolome (Ansoleaga et al., 2015; Panyard et al., 2022).

Few metabolomic studies on aging in adult humans have been conducted (Panyard et al., 2022), and most of them applied a targeted approach. In this line, large‐cohort untargeted metabolomic studies that cover a wide range of metabolites are needed to obtain robust and unexpected BM that help to propose novel modulated metabolic pathways and shed light on the mechanisms of aging. Additionally, large‐cohort studies guarantee the proper application of sex/gender perspective to assess aging, as this process is different according to sex, which, in its turn, is one of the main factors defining plasma metabolome (Jové et al. 2016; Panyard et al., 2022).

The main objective of the present study was to describe the changes in plasma metabolome associated with biological aging and the role of sex in the metabolic regulation during aging. Since the metabolome offers a comprehensive, dynamic, and precise picture of the phenotype, in this work we applied high‐throughput untargeted mass spectrometry (MS) based metabolomic techniques of a healthy adult population consisting of 1030 subjects aged ≥50 years (range 50–98 years), being 45.9% females, and 54.1% males, to systematically define hub metabolites and BM of the aging process. Furthermore, we analyzed the specificity of sex in this physiological process and validated the results using two independent cohorts (validation cohort 1: *n* = 146, 53% females, 30–100 years old; validation cohort 2: *n* = 68, 70% females, 19–107 years old).

## 2 MATERIALS AND METHODS

### 2.1 Study cohort, blood collection, and plasma isolation

The study population consisted of 1030 subjects aged ≥50 years who participated in the population‐based Aging Imageomics Study (Puig et al., 2020) from whom data were collected between November 2018 and June 2019. Eligibility criteria included age ≥50 years, dwelling in the community, no history of infection during the last 15 days, and consent to be informed of potential incidental findings. The mean age of the study population was 67.1 ± 7.3 years (range, 50–98 years), 45.9% females, and 54.1% males. Additional details on demographic and social characteristics, physical anthropometrics and health characteristics, as well as lifestyle, personality characteristics, emotional status, and cognitive function can be found in (Puig et al., 2020).

Blood samples were obtained by venipunction in the morning (between 08:00 AM and 10:00 AM) after fasting overnight (8–10 h) and collected in one VACUTAINER CPT (Cell Preparation Tube; BD) containing sodium heparin as the anticoagulant. Plasma fractions were collected after blood sample centrifugation, immediately frozen in liquid nitrogen, and transferred before 4 h to a −80°C freezer for storage at the Biobank central laboratory for future use. Due to technical issues, the number of blood samples stored to perform the metabolomic analyses was 978.

Samples and data from patients included in this study were provided by the IDIBGI Biobank (Biobanc IDIBGI, B.0000872), integrated into the Spanish National Biobanks Network and they were processed following standard operating procedures with the appropriate approval of the Ethics and Scientific Committees.

### 2.2 Plasma metabolomics analysis

For the non‐targeted metabolomics analysis, metabolites were extracted from plasma samples with methanol (containing phenylalanine‐C13 as an internal standard) according to previously described methods (Wikoff et al., 2008). Briefly, for plasma samples 30 μL of cold methanol was added to 10 μL of each sample, vortexed for 1 min, and incubated for 1 h at −20°C. Samples were centrifuged for 3 min at 12,000 *g*, and the supernatant was recovered and transferred to a chromatographic vial.

Metabolic extracts were analyzed via ultra‐high‐performance liquid chromatography (UHPLC) coupled to electrospray ionization quadrupole time of flight (ESI‐Q‐TOF) tandem mass spectrometry (MS/MS) following a previously published method (Arnoriaga‐Rodríguez et al., 2020). An Agilent 1290 liquid chromatography system (Agilent Technologies) coupled to an ESI‐Q‐TOF mass spectrometer 6545 instrument (Agilent Technologies) was used. Data were acquired in both positive and negative polarity. Samples were processed in batches with consistent quality control samples included in each batch. Two μL of the extracted sample was applied onto a reversed‐phase column (Zorbax SB‐Aq 1.8 μm 2.1 × 50 mm; Agilent Technologies) equipped with a precolumn (Zorbax‐SB‐C8 Rapid Resolution Cartridge 2.1 × 30 mm 3.5 μm; Agilent Technologies) with a column temperature of 60°C. The flow rate was 0.6 mL/min. Solvent A was composed of water containing 0.2% acetic acid, and solvent B was composed of methanol with 0.2% acetic acid. The gradient started at 2% B and increased to 98% B in 13 min and held at 98% B for 6 min. Post‐time was established in 5 min. Data were collected in positive and negative electrospray modes time of flight operated in full‐scan mode at 50–3000 m/z in an extended dynamic range (2 GHz), using N_2_ as the nebulizer gas (5 L/min, 350°C). The capillary voltage was 3500 V with a scan rate of 1 scan/s. The ESI source used a separate nebulizer for the continuous, low‐level (10 L/min) introduction of reference mass compounds 121.050873 and 922.009798, which were used for continuous, online mass calibration. MassHunter Data Analysis Software (Agilent Technologies) was used to collect the results and MassHunter Qualitative Analysis Software (Agilent Technologies) to obtain the molecular features of the samples, representing different, co‐migrating ionic species of a given molecular entity using the Molecular Feature Extractor algorithm (Agilent Technologies). We selected samples with a minimum of 2 ions. Multiple charge states were forbidden. Compounds from different samples were aligned using a retention time window of 0.1% ± 0.25 min and a mass window of 20.0 ppm ± 2.0 mDa. We selected only those present in at least 70% of the quality controls and corrected for individual bias (Broadhurst et al., 2018). The signal was corrected using a LOESS approach (Dunn et al., 2011).

### 2.3 Metabolite identification

Relevant features, expressed as mass to charge ratio and retention time, were searched against the Human Metabolome Database (HMDB) (Wishart et al., 2018), setting a mass accuracy <30 ppm and taking into account the following adducts: M + H, M + H‐H2O, M + NH4, M + Na, and M + K for positive ionization mode and M‐H, M‐H‐H2O, and M + CH3COO for negative ionization mode. Potential identities according to mass and retention time were confirmed by comparing the experimental MS/MS spectra fragmentation pattern of each feature with the experimental (if available) or predicted MS/MS spectra of the potential identities reported in public databases, using the LC–MS/MS search module of the HMDB web server, as well as the Lipidmatch and MSDIAL softwares (Sumner et al., 2007; Tsugawa et al., 2020).

### 2.4 Statistical analysis

For the statistical analysis, R software version 4.0.2 was used (R Core Team R Foundation for Statistical Computing, Vienna, 2020). Metabolite levels were log‐transformed and normalized by calculating their *z*‐scores. Associations of metabolite levels with age and their interaction with sex were evaluated using robust linear models. All models were adjusted for potential confounders associated with pathological aging and that could affect the metabolome, based on an exhaustive literature review (weight, height, body mass index, systolic and diastolic pressure, antecedents of depression, hypertension, dyslipidemia, cardiac insufficiency, diabetes, renal disease, atrial fibrillation, chronic obstructive pulmonary disease, tobacco consumption, as well as serum glucose, total cholesterol and HDL and LDL cholesterol levels) (Gonzalez‐Covarrubias et al., 2013; Gonzalez‐Freire et al., 2019; Wang et al., 2017).

*p*‐Values of the coefficients for age and its interaction with sex were obtained using a robust *F*‐Test, and 95% confidence intervals were obtained by bootstrapping. MASS package was used (https://www.stats.ox.ac.uk/pub/MASS4/).

Principal component analyses (PCA) were performed using all metabolites and using only the differentially expressed ones. The importance of age in each dimension was evaluated using robust linear models between age and each dimension and adjusting for the previously stated variables. Loadings of each metabolite for each dimension were calculated. The first 5 PC were considered for the analyses. FactoMineR (http://factominer.free.fr/) and factoextra (https://cran.r‐project.org/web/packages/factoextra/index.html) packages were used.

Interactions between metabolites were assessed using correlation and network analyses. Spearman's rank correlations were calculated between all metabolites stratifying by sex, and significant moderate or higher correlations (FDR *p*‐value < 0.05 and Spearman's rho > 0.3) were included in the respective networks. Network clusters were calculated using a multi‐level modularity optimization algorithm for finding community structure (Blondel et al., 2008). Corrplot (https://github.com/taiyun/corrplot) and igraph (https://igraph.org/) packages were used.

### 2.5 Validation of the results

Two independent cohorts were used to validate the results obtained with the discovery cohort.

#### 2.5.1 Validation cohort 1

Healthy human subjects (*n* = 146, 68 males and 78 females) with an age range from 30 to 100 years were recruited as previously described (Jové et al., 2016). All subjects were fully informed, and experimental procedures were approved by the Clinical Research Ethics Committee of the Hospital Clinico of Madrid. Plasma QTOF‐based untargeted metabolomic analyses were performed, as previously described (Jové et al. 2016).

#### 2.5.2 Validation cohort 2

Healthy human subjects (*n* = 68, 20 males and 48 females) with an age range from 19 to 107 were selected from the population data system of the 11th Health Department of the Valencian Community (Valencia, Spain), as previously described (Jové et al., 2017). All subjects were fully informed, and experimental procedures were approved by the Committee for Ethics in Clinical Research of the Hospital de la Ribera (Alzira, Valencia, Spain). Plasma QQQ‐based targeted lipidomic and metabolomic analyses were applied, as previously described (Mota‐Martorell et al., 2021; Pradas et al., 2018).

Using these validation cohorts, those metabolites and lipids that were associated with age in the discovery cohort were selected, data were log‐transformed and normalized, and robust linear models were performed in all subjects, in men or women depending on the association found in the discovery cohort. Since untargeted metabolomics is a semi‐quantitative approach and no absolute concentrations are calculated, we considered that a metabolite was validated if there was a significant association in the same direction in at least one of the validation cohorts. *p*‐Values of the coefficients were obtained using a robust *F*‐test, and 95% confidence intervals were obtained by bootstrapping.

## 3 RESULTS

### 3.1 The plasma metabolome changes with aging and has a sex‐specific regulation

An untargeted metabolomic methodology was applied to define potential BM and affected pathways. After quality control, filtering, and instrumental drift correction, 2748 metabolites from both positive and negative ionization modes were detected and included in the subsequent analyses.

**FIGURE 1:**
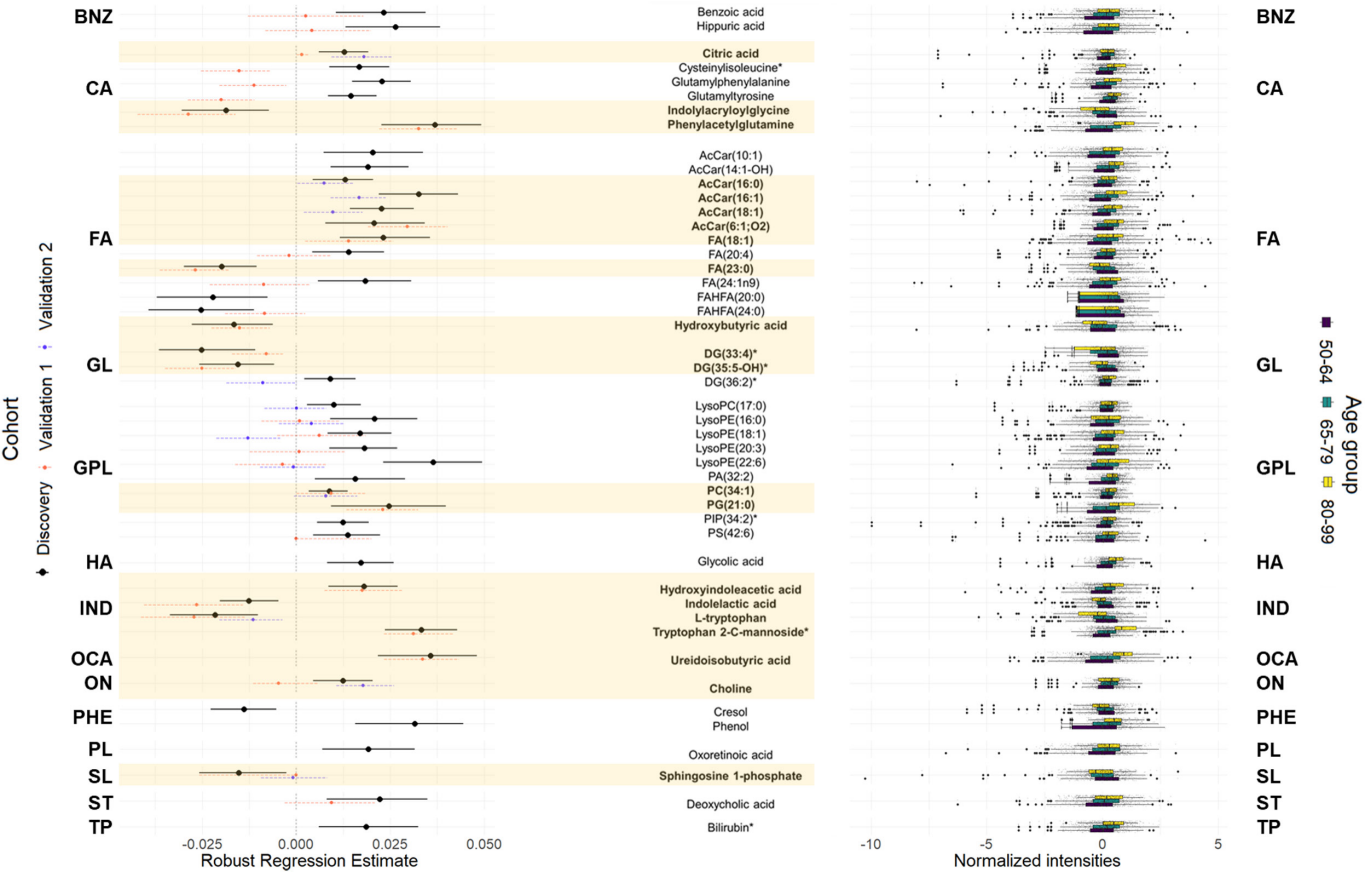
Plasma metabolic species associated with age regardless of gender grouped by class. Those features validated in independent cohorts are highlighted in bold and in yellow shade. Left panel: Forest plot of the significant (FDR *p*‐value < 0.05) robust linear regression results for the association between the relative abundance of each metabolite (log‐transformed and *z*‐scored) and age, adjusted by weight, height, body mass index, systolic and diastolic pressure, antecedents of depression, hypertension, dyslipidemia, cardiac insufficiency, renal disease, atrial fibrillation, COPD, tobacco consumption and STAI state, as well as serum glucose, total cholesterol and HDL and LDL cholesterol levels in the Discovery cohort. Dots represent the robust linear regression estimates for age, and the whiskers represent bootstrapped 95% confidence intervals. Colors represent the cohort used for the regression (Black: Discovery, Orange: Validation cohort 1, Blue: Validation cohort 2). Eleven negative correlations (8 of them validated) and 36 positive correlations (13 of them validated) were found. Right panel: Boxplots of the metabolites stratified by age groups (purple: 50–64 years old, blue: 65–79 years old, yellow: 80–99 years old). AcCar, Acylcarnitine; BNZ, Benzene and substituted derivatives; CA, Carboxylic acids and derivatives; DG, Diacylglyceride; FA, Fatty Acyls; FAHFA, Fatty Acyl Esters of Hydroxy Fatty Acid; GL, Glycerolipids; GPL, Glycerophospholipids; HA, Hydroxy acids and derivatives; IND, Indoles; OCA, Organic carbonic acids and derivatives; ON, Organonitrogen compounds; PA, Phosphatidic acid; PC, Phosphocholine; PE, Phosphoethanolamine; PG, Phosphoglycerol; PHE, Phenols; PIP, Phosphoinositol phosphate; PL, Prenol lipids; PS, Phosphoserine; ST, Steroids and steroid derivatives; TP, Tetrapyrroles and derivatives. All metabolites were identified based on exact mass, retention time and MS/MS spectrum, except those with (*) that were only identified based on exact mass and retention time.

In order to evaluate the effect of aging on the metabolome, robust linear models were performed considering age, sex, and its interaction with age. These models revealed 137 features (47 of them identified) that were significantly associated with age regardless of sex (FDR adjusted *p*‐value < 0.05). Among those features, 43 of them showed a negative association with age and 94 showed a positive association (Dataset S1). The identified compounds belonged to the following classes: 2 benzenes, 6 carboxylic acids, 13 fatty acyls, 3 glycerolipids, 10 glycerophospholipids, 1 hydroxy acid, 4 indoles, 1 organic carbonic acid, 1 organonitrogen compound, 2 phenols, 1 prenol lipid, 1 sphingolipid, 1 seteroid, and 1 tetrapyrrole. Among the 47 identified compounds, 37 were detected in the independent cohorts and 21 of the detected metabolites (57%) were validated (Figure 1).

**FIGURE 2:**
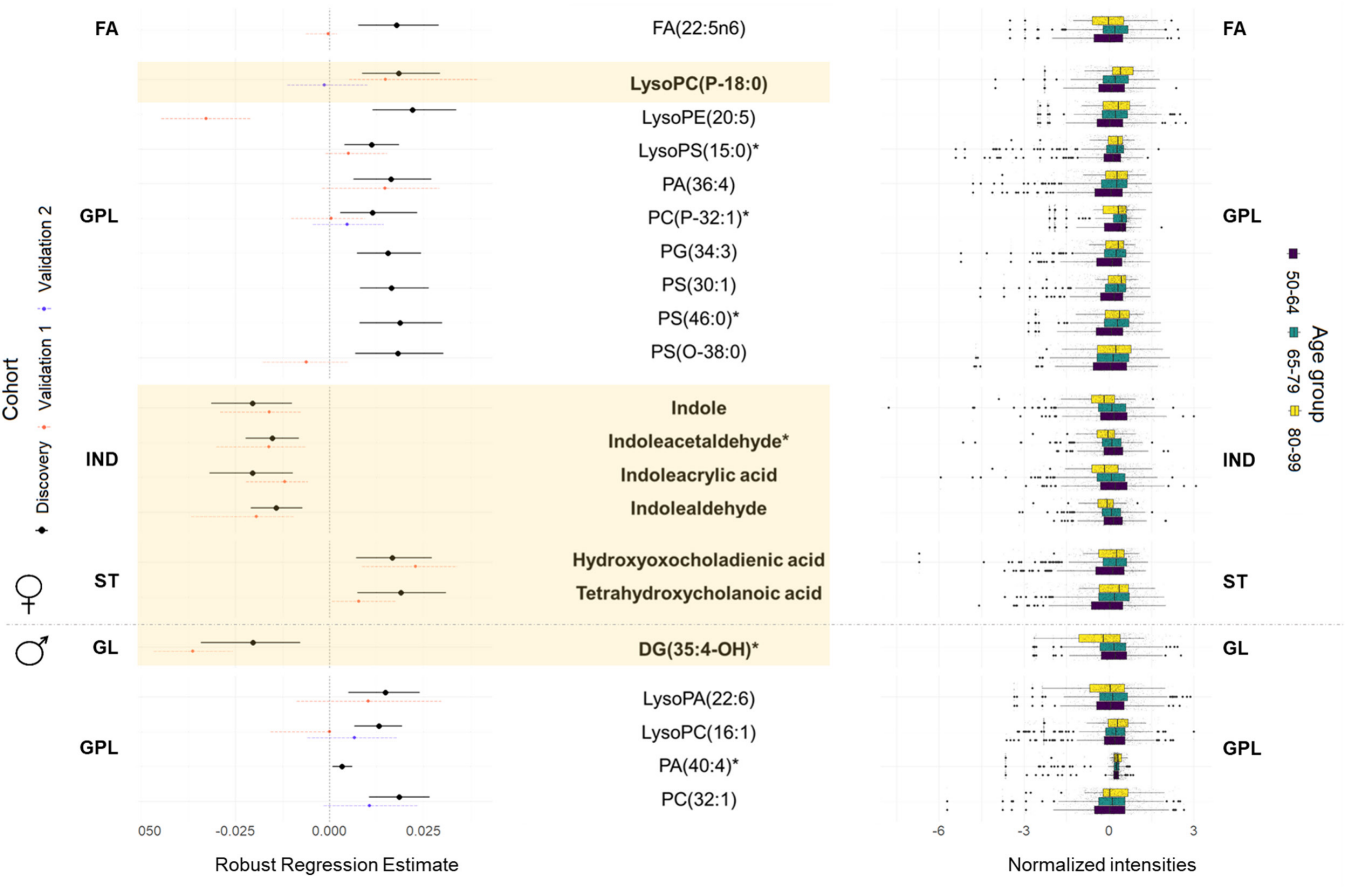
Gender‐specific plasma metabolic species associated with age. Those features validated in independent cohorts are highlighted in bold and in yellow shade. Left panel: Forest plot of the significant (FDR *p*‐value < 0.05) robust linear regression results for the association between the relative abundance of each metabolite (log‐transformed and *z*‐scored) and age for each gender, adjusted by weight, height, body mass index, systolic and diastolic pressure, antecedents of depression, hypertension, dyslipidemia, cardiac insufficiency, renal disease, atrial fibrillation, COPD, tobacco consumption and STAI state, as well as serum glucose, total cholesterol and HDL and LDL cholesterol levels in the Discovery cohort. Dots represent the robust linear regression estimates for age, and the whiskers represent bootstrapped 95% confidence intervals. Colors represent the cohort used for the regression (Black: Discovery, Orange: Validation cohort 1, Blue: Validation cohort 2). Four negative correlations (all of them validated) and 12 positive correlations (3 of them validated) were found in women, and 1 negative correlation (validated) and 4 positive correlations (none of them validated) were found in men. Right panel: Boxplots of the metabolites stratified by age groups (purple: 50–64 years old, blue: 65–79 years old, yellow: 80–99 years old). DG, Diacylglyceride; FA, Fatty Acyls; GPL, Glycerophospholipids; IND, Indoles; PA, Phosphatidic acid; PC, Phosphocholine; PE, Phosphoethanolamine; PG, Phosphoglycerol; PS, Phosphoserine; ST, Steroids and steroid derivatives. All metabolites were identified based on exact mass, retention time and MS/MS spectrum, except those with (*) that were only identified based on exact mass and retention time.

Regarding sex, we found a set of 71 metabolites with a sex‐specific regulation, which was associated with age only in men (12, 5 of them identified) or in women (59, 16 of them identified) (Dataset S1). In men, we identified 1 glycerolipid and 4 glycerophospholipids. In women, we found 1 fatty acyl, 9 glycerophospholipids, 4 indoles, and 2 steroids. Four of the identified compounds in men and 13 in women were detected in independent cohorts, and 1 (25%) and 7 (54%) of them were validated, respectively (Figure 2).

**FIGURE 3:**
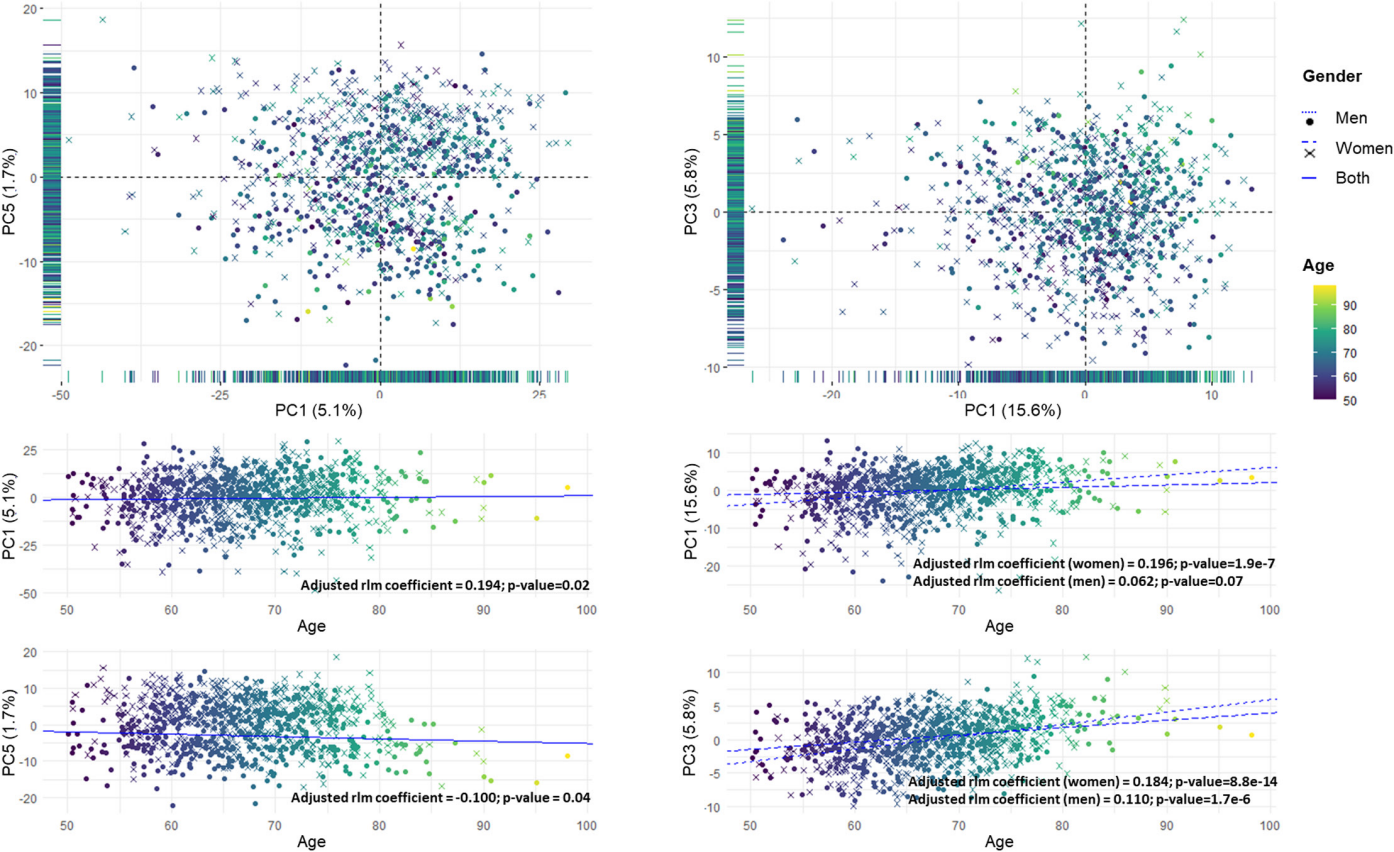
Principal component analysis of all the individuals from the study cohort. Top panel: Scores plot using all the metabolites (left) and using only those plasma metabolites significantly associated with age (right). The principal components with highest association with age are represented. The projection of the scores plot in only one component is represented in its corresponding axis. Bottom panel: Association between each principal component and age. Each dimension is represented in the *x*‐axis, and age is represented in the *y* axis. The lines represent the fitted robust linear model for the association between age and each component. A single solid regression line is represented in the cases in which there is no significant interaction between age and gender. Two regression lines are represented when there is a significant interaction, using dotted lines men and dashed lines for women. Samples are colored by age, from 50 years old (purple) to 99 years old (yellow). Men are represented using dots and women using crosses.

To further obtain a global overview of the changes in the metabolome induced by biological aging, unsupervised multivariate statistics were applied. This approach revealed that age is one of the main relevant sources of variability in the whole metabolome. When applying a PCA to the whole dataset and considering the first 5 dimensions (which together represent 15.1% of the total variance), we observed that the first, the fourth, and the fifth principal components, which, respectively, represent the 5.1%, 1.9%, and 1.7% of the total variance, were significantly associated with age after adjusting for confounders (Figure 3). Furthermore, 7 of the top 10 contributing metabolites to the first dimension (70%) and 78 of the 449 metabolites with a higher‐than‐expected contribution to dimension 1 (17%) were associated with age. The list of contributions of each metabolite to the first 5 dimensions of the PCA is reported in the Dataset S1. Based on the total number of metabolites included in the PCA, the expected contribution of each metabolite to each component is 0.036, calculated as 100/number of metabolites. The separation of individuals according to age in the PCA is enhanced when using only the metabolites significantly associated with age. In this case, the first five components represented 35% of the total variance, and age was associated with all five components (Figure 3).

### 3.2 Lipid and aromatic amino acid metabolism are the main metabolic pathways affected by age

Among the 208 differential metabolites (137 associated with age regardless of sex and 71 with a sex‐specific regulation), 68 (33%) have a potential identity based on exact mass, retention time, isotopic distribution, and/or MSMS spectra. In Figure S1, all the differential metabolites (both identified and unidentified) are represented according to their mass to charge ratio and retention time in order to obtain an overview of their physicochemical properties. Based on the identities of these metabolites, we can state that lipid metabolism has an important impact on the aging process: 46 of the 68 (68%) total identified molecules are lipids. Regarding lipid species, several lipid families related to bioenergetics (glycerolipids and fatty acyls), structural (glycerophospholipids) and signaling (sphingolipids, glycerophospholipids, glycerolipids, prenol lipids, and steroids) functions are affected. It is important to note that the methodology applied is not suitable to detect the most nonpolar species, such as triglycerides and cholesteryl esters.

Remarkably, we found 6 acylcarnitines (AcCar), 4 of them long‐chain AcCar, that were positively associated with age. Four unsaturated long‐chain fatty acyls FA(22:5n6) (docosapentaenoic acid, DPA) in women, and FA(20:4n6) (arachidonic acid, AA), FA(18:1 n9) (oleic acid, OA), and FA(24:1n9) (nervonic acid, NA) in all subjects had also a positive association with aging whereas the saturated long‐chain fatty acid FA(23:0) was negatively associated with age. Among glycerophospholipids, all of them were positively associated with age, either in all subjects or for a specific sex. It is important to remark that 10/23 (43%) were lysophospholipids which are related to membrane remodeling and cell signaling. In the same line, the non‐lipid specie choline, which is a precursor of glycerophospholipid and sphingolipid biosynthesis, but also the precursor of the neurotransmitter acetylcholine, was positively associated with age. Contrarily, the bioactive lipid sphingosine‐1‐P presented a negative association with age.

**FIGURE 4:**
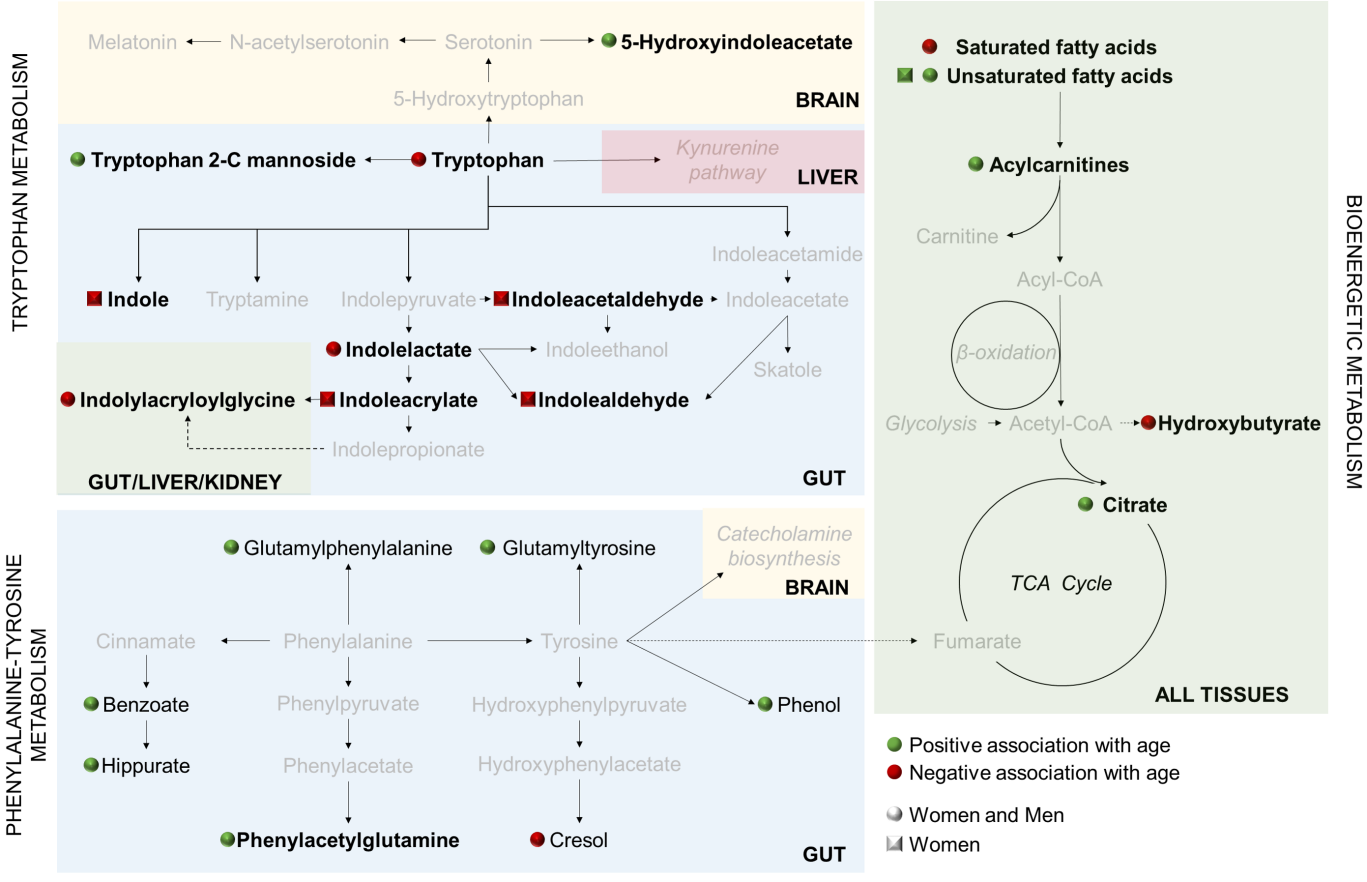
Main metabolic pathways affected by age. Metabolites with significant associations with age are represented in black, and those without significant associations are represented in grey. Metabolic pathways are written in italics. Squares near the metabolites indicate that those features associated with a age specifically in women, and spheres indicate that these metabolites are associated with age in both men and women. Green fill indicates positive association, and red fill indicates negative association. Solid arrows indicate that the metabolite is obtained from a direct enzymatic reaction, and dashed arrows represent that there are intermediate metabolites in the pathway. Each background color represents a tissue; Blue: Gut (gut microbial pathways); Green: Multiple tissues; Red: Liver; Yellow: Brain. Those metabolites validated in independent cohorts are highlighted in bold.

Regarding the non‐lipid species, metabolites belonging to microbial and human aromatic amino acid (AAA) metabolism are the most representative (16 of 22, 73%). Specifically, 7 compounds belonged to the tyrosine‐phenylalanine gut microbial metabolism (benzoic acid, cresol, glutamylphenylalanine, glutamyltyrosine, hippuric acid, phenol, and phenylacetylglutamine) and all of them except cresol were positively associated with age. Among the remaining 9 metabolites, which were related to the tryptophan metabolism, 7 of them were related to gut microbiota and were negatively associated with age (indole, indoleacetaldehyde, indoleacrylic acid and indolealdehyde in women and indolelactic acid, indolylacryloylglycine and tryptophan in all subjects) and 2 of them were positively associated with age in all subjects (tryptophan‐C‐mannoside and hydroxyindoleacetic acid). The related pathways are represented in Figure 4. Furthermore, the secondary bile acid deoxycholic acid, also related to gut microbiota, was positively associated with age. Finally, glycolic acid was positively associated with the aging process.

The results regarding acylcarnitines and tryptophan‐related metabolites were consistently validated. On the contrary, the validation of the results of tyrosine‐phenylalanine, fatty acid, and glycerolipid‐related metabolites was less consistent and more studies are needed to clarify the specific dysregulation of these pathways during aging.

### 3.3 Specific metabolic clusters are affected by age

**FIGURE 5:**
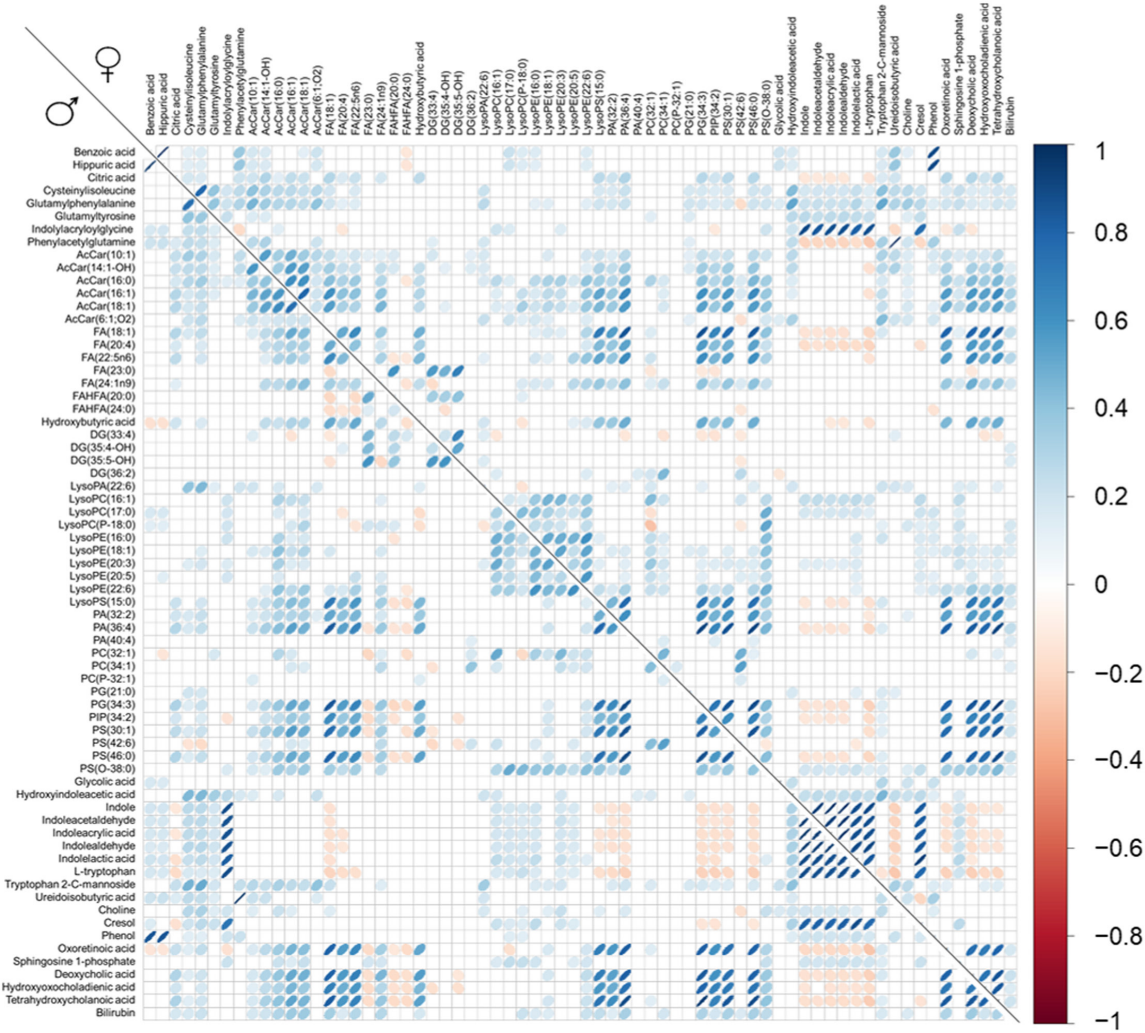
Correlations plot for all the identified plasma metabolic species associated with age. Statistically significant (FDR *p*‐value < 0.05) Spearman's rank correlations between each pair of metabolites are represented stratifying by gender (men: lower diagonal, women: upper diagonal). Positive correlations are represented in blue and negative correlations in red.

In order to discern the sex‐specific metabolite regulation associated with the differential features, inter‐metabolite relationships were assessed by applying correlation and network analyses for each sex to all the age‐associated metabolites. Globally, strong Spearman correlations between metabolites were similar in women and men, and only those with the lowest correlation coefficients were sex‐specific (Figure 5). For correlation coefficients, see the Dataset S1. Interestingly, this analysis revealed the existence of specific groups of metabolites with strong intra‐group correlations.

**FIGURE 6:**
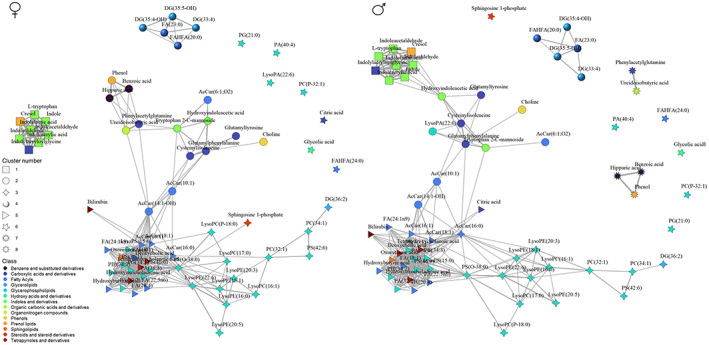
Network analysis of the identified plasma metabolites associated with age stratifying by gender. Medium and strong correlations (FDR *p*‐value < 0.05, Spearman's rho > 0.3) have been used. Each metabolite is represented as a node, colored according to its metabolic class and shaped according to its assigned cluster using a multi‐level modularity optimization algorithm. Each correlation is represented as an edge and its width proportional to the correlation coefficient.

To further explore this point, network analyses using moderate and strong correlations (FDR *p*‐value < 0.05 and Spearman's rho > 0.3) and network clusters were calculated (Figure 6). Specifically, 8 clusters were identified in men and 6 in women (Table 1). Globally, there are two non‐lipid clusters (clusters 1 and 2) and three lipid clusters (clusters 3, 4, and 5). Cluster 6 includes features without moderate or strong correlations with any other feature. In men, 2 extra non‐lipid clusters appear (clusters 7 and 8) which are independent of the rest of the clusters and from each other.

**TABLE 1:** Clusters of inter‐related metabolites.

Cluster 1	Cluster 2	Cluster 3	Cluster 4	Cluster 5	Cluster 6	Cluster 7	Cluster 8
*Both genders*	*Both genders*	*Both genders*	*Both genders*	*Both genders*	*Both genders*	*Men*	*Men*
Indolylacryloylglycine	Cysteninylisoleucine	DG(36:2)	FA(23:0)	AcCar(16:1)	FAHFA(24:0)	Benzoic acid	Phenylacetylglutamine
Indole	Glutamylphenylalanine	LysoPC(16:1)	FAHFA(20:0)	AcCar(18:1)	PA(40:4)	Hippuric acid	Ureidoisobutyric acid
Indoleacetaldehyde	Glutamyltyrosine	LysoPC(17:0)	DG(33:4)	FA(18:1)	PC(P‐32:1)	Phenol	
Indoleacrylic acid	AcCar(10:1)	LysoPC(P‐18:0)	DG(35:4‐OH)	FA(20:4)	PG(21:0)		
Indolealdehyde	AcCar(14:1‐OH)	LysoPE(16:0)	DG(35:5‐OH)	FA(22:5n6)	Glycolic acid		
Indolelactic acid	AcCar(6:1:O2)	LysoPE(18:1)		FA(24:1n9)	*Women*		
L‐tryptophan	Hydroxyindoleacetic acid	LysoPE(20:3)		Hydroxybutyric acid	Citric acid		
Cresol	Tryptophan 2‐C‐mannoside	LysoPE(20:5)		LysoPS(15:0)	LysoPA(22:6)		
	Choline	LysoPE(22:6)		PA(32:2)	*Men*		
	*Women*	PC(32:1)		PA(36:4)	Sphingosine 1‐phosphate		
	Benzoic acid	PC(34:1)		PG(34:3)			
	Hippuric acid	PS(42:6)		PIP(34:2)			
	Phenylacetylglutamine	PS(O‐38:0)		PS(30:1)			
	Ureidoisobutyric acid	*Women*		PS(46:0)			
	Phenol	Sphingosine 1‐phosphate		Oxoretinoic acid			
	*Men*	*Men*		Deoxycholic acid			
	LysoPA(22:6)	AcCar(16:0)		Hydroxyoxocholadienic acid			
				Tetrahydroxycholanoic acid			
				Bilirubin			
				*Women*			
				AcCar(16:0)			
				*Men*			
				Citric acid			

Abbreviations: AcCar, Acylcarnitine; DG, Diacylglyceride; FA, Fatty Acyls; FAHFA, Fatty Acyl Esters of Hydroxy Fatty Acid; PA, Phosphatidic acid; PC, Phosphocholine; PE, Phosphoethanolamine; PG, Phosphoglycerol; PIP, Phosphoinositol phosphate; PS, Phosphoserine.

Clusters 1 and 2 include all non‐lipid species except bilirubin, citric acid, and glycolic acid. Cluster 1 includes cresol and all the metabolites related to tryptophan gut microbial metabolism except Tryptophan 2‐C mannoside, and cluster 2 includes the rest of the non‐lipid species and the shorter chain AcCar. Interestingly, in men, most of the phenylalanine and tyrosine related metabolites are included in the men‐specific clusters 7 and 8 and not in cluster 1.

Regarding lipid clusters, cluster 3 is mainly a phospholipid cluster and includes all lysophosphatidylethanolamines (lysoPE) and lysophosphatidylcholines (lysoPC), as well as some phsophatidylcholines (PC) and phosphatidylserines (PS). Cluster 4 includes mainly DG, and cluster 5 includes hydroxybutyric acid, some glycerophospholipids, the longer chain AcCar, all long‐chain unsaturated fatty acids and all the steroids and prenol lipids.

It is noteworthy that although cluster 1 is common in both sex, it interacts differently with other clusters: while it is independent of other clusters in women, in men is related to cluster 2 through hydroxyindoleacetic acid. Five metabolites from cluster 1 (cresol, indole, indoleacrylic acid, indolealdehyde, and indolelactic acid) are correlated with hydroxyindoleacetic acid in men. Interestingly, 3 of these molecules (indole, indoleacrylic acid, indolealdehyde) were identified as women‐specific for aging. Furthermore, metabolites related to phenylalanine and tyrosine metabolism (hippuric acid, benzoic acid, phenol, and phenylacetylglutamine), together with ureidoisobutyric acid, show also important sex differences regarding their relationships with other metabolites. Interestingly, when the analyses were performed using only those features validated in an independent cohort, we obtained very similar results (Figure S2).

## 4 DISCUSSION

Aging biology has been extensively studied during decades for its direct relation to the determination of longevity and its principal role in the development of age‐related diseases, which are responsible for the major disabilities all over the World. The study of this universal process requires the effort of many researchers in gerontology and the application of different approaches and techniques to achieve an integrated view from molecular to organismal level. In this sense, the application of high‐throughput techniques to large populations is essential to describe novel BM of aging, helping to better understand the underlying biological mechanisms to achieve a healthy aging and to prevent the development of age‐related diseases.

In the present work, we used a well‐characterized cohort of 1030 healthy human adults (45.9% females and 54.1% males) ranging from 50 to 98 years to detect plasma hub metabolites and BM of aging including a sex/gender perspective using an MS‐based untargeted metabolomic approach. With the objective of defining BM of biological aging, the results were adjusted for previously described potential confounders associated with pathological aging (Gonzalez‐Covarrubias et al., 2013; Gonzalez‐Freire et al., 2019; Wang et al., 2017). Importantly, two independent cohorts were used for validating the results (validation cohort 1: *n* = 146, 53% females, 30–100 years old; validation cohort 2: *n* = 68, 70% females, 19–107 years old).

The analyses revealed that aging is accompanied by significant changes in the metabolome that affect several metabolites and lipid classes. Globally, plasma metabolome is able to discriminate the individuals according to their age suggesting a specific metabolic signature of the aging process. These changes mostly involve lipid metabolism‐related metabolites, which is foreseeable if we consider that more than 80% of the metabolome is composed of lipid species (Wishart et al., 2009), as well as human and gut microbial AAA metabolism‐related metabolites. Importantly, although there are global changes associated with aging, there is a sex‐specific signature that it is crucial to consider in order to better understand this process. Specifically, whereas 59 metabolites are associated with age specifically in women, only 12 are associated in men. Interestingly, among the identified species, all the lipid species specifically affected by age in women increased with age whereas the indoles decreased. In men, all the identified metabolites affected are lipids and only one, DG(35:4‐OH) is negatively associated with aging. These results reinforce the importance of applying a sex/gender perspective approach when the age‐associated changes in metabolome are evaluated.

Lipids are a diverse class of compounds involved in multitude of structural and functional properties related to cell membranes, signaling roles, and bioenergetics. In the present work, concerning the lipid‐related molecules affected regardless of sex, we found 6 AcCar (4 with a long‐chain and 2 with a medium chain fatty acid in their structure) that were positively associated with aging. The main function of AcCar is to ensure fatty acid transport into the mitochondria for β‐oxidation and energy production (Reuter & Evans, 2012). Thus, an increased concentration of plasma AcCar with aging may represent an adaptive response to increased bioenergetic demands with age, a dysfunction in fatty acid oxidation and overall mitochondrial activity, or both. A previously published lipidomic study performed with 980 individuals aged 18–87 years old revealed no significant changes in total intensity of AcCar with age and sex (Slade et al., 2021). However, several studies described a positive association of plasma levels of specific AcCar with aging and the impact of sex in these correlations (Jarrell et al., 2020; Johnson et al., 2019; Takiyama & Matsumoto, 1998). Importantly, a lipidomic study performed with 10.339 participants described that the majority of AcCar were significantly higher in men and positive associations of age with AcCar, being the AcCar(14:2) the one with the strongest association (Beyene et al., 2020). Interestingly, previous studies in cell cultures demonstrated that various medium‐chain AcCar, including the AcCar(10:1), that is positively associated with age in the present study, induces lipid peroxidation in cells from rat frontal cortex contributing to generate a more pro‐oxidative status (Tonin et al., 2012). The higher presence of this medium‐chain AcCar in plasma of aged individuals may be related to the increased oxidative stress‐derived damage associated with the aging process. In line with this, other authors suggested increased levels of AcCar in several age‐related pathologies, mainly cardiovascular and neurodegenerative diseases (Gao et al., 2021; Horgusluoglu et al., 2022; Kukharenko et al., 2020; Liu et al., 2018; Toledo et al., 2017) but also as BM of frailty in elderly subjects (Malaguarnera et al., 2020). However, other studies described an inverse relationship with these conditions (Ciavardelli et al., 2016; Shao et al., 2020).

In addition to AcCar, we also found a positive correlation between age and 4 unsaturated long‐chain fatty acids. Globally, it has been described that plasma saturated, polyunsaturated and monounsaturated fatty acids increase with age (Johnson & Stolzing, 2019). Specifically, our results are partially supported by previously published data (De Sanctis et al., 2020). The differences could be attributed to the fact that most of the previously published studies are performed with discrete age groups rather than using age as a continuous factor. We found that DPA (22:5n6) was associated with age only in women, suggesting a different regulation of biosynthesis pathway with age according to sex. This differential regulation has been suggested previously by other authors (Zhao et al., 2020). It is important to note that DPA is a minor fatty acid and more research is needed to understand its role in tissue/cell metabolism and its function in physiopathological processes. AA and its metabolites (eicosanoids) are extensively related to pro‐inflammatory events (Das, 2018). It is previously stated that the low‐grade inflammation described in age‐related diseases is caused, almost partially, by favoring the production of pro‐inflammatory species from AA (Das, 2018). Furthermore, the polyunsaturated nature of these fatty acids makes them more prone to oxidative damage (Mota‐Martorell et al., 2022), so the increased levels of both AA and DPA observed with age suggest a favored environment to inflammation and oxidative stress in tissues with age, as previously described (de Almeida et al., 2020). This hypothesis should be confirmed applying specific methodology to detect inflammatory intermediates and oxidative stress derivates.

Elevated levels of monounsaturated fatty acids in plasma have been previously related to decreased peroxisomal function, a symptom of age‐related diseases (Yamazaki et al., 2014). NA (FA(24:1n9)) is a long‐chain monounsaturated fatty acid that is enriched in sphingomyelin and may enhance brain functions and promotes myelin synthesis (Li et al., 2019). Altered levels of NA have been previously described in age‐related diseases and peroxisomal β‐oxidation disorders (Oda et al., 2005; Sargent et al., 1994; Yamazaki et al., 2014) but, as far as we know, this is the first time that this fatty acid is evaluated in aging. Plasma high levels of OA (FA(18:1n9)) have been previously associated with health status. Contrarily, lower levels of this monounsaturated fatty acid have been related to age‐associated neurodegenerative (Cunnane et al., 2012) and cardiovascular diseases (Lai et al., 2018; Terés et al., 2008). Specifically, a previously published paper pointed out OA as a sensor for fatty acid oxidation to maintain lipid homeostasis (Lim et al., 2013). In line with this, the higher levels of OA observed with age could be an adaptive response to β‐oxidation impairment.

In the present work, we found and validated that the final product of β‐oxidation hydroxybutyrate is decreased with aging, reinforcing the idea of impaired mitochondrial β‐oxidation. It is important to remark that the levels of the tricarboxylic acid (TCA) cycle intermediate, citrate, were consistently increased with age in all cohorts (Mota‐Martorell et al., 2021). Assuming that mitochondrial β‐oxidation is decreased with age, this increase could be provoked by higher glycolytic activity of the cells. This conclusion is reinforced by network analyses where long‐chain fatty acids, long‐chain AcCar, and hydroxybutyric acid clustered together, as well as citrate in men (cluster 5). In this sense, a previous study has described that a citrate‐based ketogenic‐promoting diet enhances metabolic health and longevity (Fan et al., 2021). Interestingly, glycolic acid, previously related to bioenergetics (Maniscalco et al., 2017), was positively associated with the aging process.

All these results suggest that aging is accompanied by an impairment of mitochondrial fatty acid oxidation, resulting in accumulation of intracellular AcCar and long‐chain fatty acyls and a decrease in the final product of β‐oxidation hydroxybutyrate (Schooneman et al., 2013), which is subsequently reflected in plasma (Huynh et al., 2019). The increased levels of citrate could be explained by an increase in glycolysis pathway caused by the β‐oxidation impairment. Mitochondrial dysfunction is considered one of the major hallmarks of aging and alterations in TCA cycle metabolites (specifically citrate) and FA were previously reported by other authors as indicators of a reduction of mitochondria effectiveness resulting in energy production dysfunction favoring increased levels of oxidative stress (Panyard et al., 2022).

Glycerophospholipids are important structural and functional components of biological membranes. In the present work, we described several types of glycerophospholipids; all of them positively correlated with age. Interestingly, there is an important sex‐specific effect of aging in this lipid category. Among the glycerophospholipid described, we found several lysophopholipids, with a greater predominance of LysoPE. Lysophoshpolipids are generated from phospholipids by the action of phospholipases and are mainly related to lipid remodeling and cell signaling functions (Hishikawa et al., 2014). Regarding the LysoPE described in the present study, LysoPE(18:1) has been previously related to obese individuals (Wang et al., 2016) and it is previously described that the levels of LysoPE(22:6) and LysoPE(18:1) are increased in diabetic individuals (Wang et al., 2016). As far as we know, there is no previous information about the effect of age and sex on the specific species described in this work.

Sphingosine‐1‐P is a bioactive sphingolipid implicated in numerous aspects of cell physiology, including cell survival and inflammatory responses, and it has been directly associated with neuroprotective effects (Czubowicz et al., 2019). Furthermore, alterations in sphingosine‐1‐P levels were described in brain tissue and serum of individuals suffering from age‐related neurodegenerative and cardiovascular diseases (Velazquez et al., 2021). Our results (validated in independent cohorts) indicated that the levels of this sphingolipid decreased with age in both women and men, although some papers indicated sex‐specific regulation in both plasma (Guo et al., 2014) and brain tissue (den Hoedt et al., 2021) in human beings and animal models. The negative correlation of sphingosine‐1‐P levels with aging could explain, at least partially, the physiological cognitive decline characteristic of the aging process.

The family of FAHFA are signaling molecules that have been previously associated with anti‐inflammatory and anti‐diabetic properties (Brejchova et al., 2020). In the present work, two saturated FAHFA (FAHFA(20:0) and FAHFA(24:0)) were negatively associated with age. There is still little information about specific FAHFA in bibliography, and most of the studies were focused on FAHFA composed by FA(16:0), FA(16:1), FA(18:0), FA(18:1), and FA(18:2). Specifically, it has been previously described that these species can be modulated by the aging process in both plasma and tissue (Kellerer et al., 2021; Zhu et al. 2020). As far as we know there is no available information about the specific species described in the present work.

The changes in gut microbiota during life could be used as a BM of chronological aging (Galkin et al., 2020). Interestingly, gut‐derived bacterial metabolites, including AAA and their derived catabolites, have been previously related to human health and disease (Tran & Hasan Mohajeri, 2021). The AAA‐derived metabolites have been previously described as important players in the gut–brain axis (Tran & Hasan Mohajeri, 2021) and have been related to memory scores in humans and animal models (Arnoriaga‐Rodríguez et al., 2020; Noristani et al., 2012). AAA catabolites by the gut microbiome may regulate immune, metabolic, and neuronal responses at local and distant sites and regulate inflammatory processes in different tissues including liver, kidney heart, and brain (Roager & Licht, 2018). Regarding tryptophan metabolism, an association with age was found and validated in 9 metabolites (4 of them were associated only in women). The role of tryptophan and its indole derivatives in memory processes (Arnoriaga‐Rodríguez et al., 2020; Roager & Licht, 2018) and neurodegenerative diseases (Tran & Hasan Mohajeri, 2021) has been previously described by other authors. Interestingly, the relationship between alterations in tryptophan metabolism and memory described in a previous work using the same cohort as the present study (Arnoriaga‐Rodríguez et al., 2020) was only observed in obese individuals, and the authors associated these changes with the obesity‐derived inflammatory status. In the present work, we found that both tryptophan and the reported indole catabolites produced by gut microbiota were negatively associated with age, which could be associated with the physiological decline of nervous system observed with age. Interestingly, this catabolic pathway is more affected in women suggesting sex‐specific changes in gut microbiota during aging. These results could partially explain the higher incidence rates of AD and dementia described in women, which are estimated to be 2:1 compared with men (Ferretti et al., 2020). The tryptophan‐related metabolite Tryptophan‐C‐mannoside, which has also been previously associated with cognitive declines (Rong et al., 2021), has been positively associated with aging. Additionally, the serotonin pathway (brain) from tryptophan seems to be enhanced to produce higher levels of 5‐hydroxyindoleacetate. Concerning tryptophan metabolism, previous work performed in mice liver demonstrated the affectation of kynurenine pathway after applying an “anti‐aging” nutritional intervention such as 30% caloric restriction (Jové, Naudí, et al., 2014). No affectation of this pathway was observed in the present work.

Regarding phenylalanine and tyrosine‐derived metabolites produced by gut microbiota, we found 6 metabolites positively associated with age and only one (cresol) with a negative association. Among these metabolites, cresol levels have been previously related to neurodegeneration and age‐related diseases (Tran & Hasan Mohajeri, 2021), and the levels of phenylacetylglutamine have been previously associated with AD (González‐Domínguez et al., 2016). The levels of hippuric acid positively correlate with the aging process in our work. Other authors obtained the same results and have also related this metabolite to cognitive decline (De Simone et al., 2021), and have been also related to cognitive decline by other authors (De Simone et al., 2021). The different regulation of gut‐derived AAA catabolism in women and men is reinforced by correlational and network analyses where it is shown an independent cluster (cluster 1) in women whereas in men those metabolites have stronger connections with cluster 2. Interestingly, a recent review described that a decrease in plasma levels of tryptophan and the increase of tyrosine with aging are one of the more consistently reported changes when studying aging metabolomics (Panyard et al., 2022).

Finally, the levels of choline were positively associated with the aging process, as previously described (Mota‐Martorell et al., 2021) and could be validated in an independent cohort. Choline metabolite has several functions in cell metabolism. First, is a precursor of glycerophopholipid and sphingolipid biosynthesis. Second, is a precursor of neurotransmitter acetylcholine. Third, choline can also be metabolized to betaine, a key methyl donor in the one‐carbon metabolism and modulator of homocysteine status. Interestingly, recently published results pointed out the importance of choline and homocysteine metabolism in defining plasma metabolome of long‐lived humans (Mota‐Martorell et al., 2021). In this sense, more studies are needed to elucidate the role of choline in the aging biology.

The main strengths of the present study are (i) the associations of the metabolites with age reported in the discovery cohort were controlled for the main potentially confounding health conditions. Confounding is one of the main issues when studying aging processes because of the interconnection between aging and several factors, but most of the available studies only adjust for sex and BMI (Panyard et al., 2022) and (ii) a large discovery cohort and two independent validation cohorts were used. These three cohorts were recruited from different regions of Spain and were analyzed using different methodological approaches. Metabolomics has inherent handicaps such as the high variable‐to‐sample ratio and the high variability of the metabolite levels and the results, which require large sample sizes and an efficient dimensionality reduction, as well as the use of validation cohorts to improve the robustness and replicability of the results. Importantly, a relevant part of the main results found in the discovery cohort could be consistently confirmed using the validation cohorts. The discrepancies observed in the validation cohorts (mainly in lipid metabolism) could be partially ascribed to the fact that in these cohorts we did not have the information regarding the potential confounders that were used in the discovery cohort, such as dyslipidemia or cholesterol levels, which have an important association with lipid metabolism. Additionally, the metabolome of the validation cohorts was analyzed using different approaches and equipment. In order to validate this part of the results, further studies focused on lipid metabolism, using specific lipidomic techniques and controlling for the proposed confounders should be performed. From our findings, several key questions remain unanswered and should be elucidated in future works: (i) we do not know whether the age‐dependent metabolic changes observed are the cause or consequence (adaptation or dysregulation) of aging, (ii) we do not know how regulatory networks change with age and how they influence or determine the aging process itself, (iii) we do not know how the different tissues are affected and the degree of participation and responsibility for the plasma metabolic profile observed, (iv) we have been able to annotate 47 of the 137 (34%) statistically molecular features. Although the annotation is a well‐known limitation of untargeted metabolomic approaches, it is important to have in mind that the identification of these metabolites could change or modify the conclusions withdrawn at the biological and mechanistic levels, (v) although we describe gut‐related metabolites, we do not know the real implication of gut microbiota in this specific age‐associated plasma metabolome, and (vi) because we analyzed the global metabolome, the changes observed in lipid species could not be ascribed to a specific lipoprotein or to their metabolism. To clarify this point, an alternative study should be assessed. Finally, and importantly, the LC/MS approach used captures a global snapshot of the organism's metabolic state but has limitations since no single analytical platform can cover the entire metabolome basically due to the great diversity of compounds and physicochemical properties.

In conclusion, it is suggested that aging induces changes in plasma metabolome that may reflect the physiological changes that occur in tissues. Our findings indicate that these changes are sex‐specific and the need to address the studies of aging from a sex/gender perspective. Specifically, we describe changes in bioenergetic pathways that point to a decrease in mitochondrial β‐oxidation and an accumulation of unsaturated fatty acids and acylcarnitines. These changes may be the substrate to the increment of oxidative stress and inflammation characteristic of this physiological process. Furthermore, we described, for the first time, the importance of gut‐derived AAA catabolites in the aging process describing novel BM that could contribute to better understand this physiological process but also age‐related diseases. The results obtained bring us closer to the description of the phenotype of this universal process and are essential to understand the physiopathology of aging.

## AUTHOR CONTRIBUTIONS

Conceptualization: J. M. F‐R., J. P., M. J., and R. P.; methodology: M. J. and R. P.; software, and formal analysis: J. S (J. Sol)., E. O., N. M‐M., I. P., J. D. G‐L., M. M‐G., A. F‐B., M. P‐O., M. J., and R. P.; investigation: M. P‐O., M. J., and R. P.; statistical analysis: J. S (J. Sol)., M. J., and R. P.; resources: M. O‐B., J. M‐P., J. V., C. B (C. Borrás)., M. dF., I. M., V. P., C. B (C. Biarnes)., P. D‐I‐E., S. T‐H., S. P., J. C. V., R. B., R. R., J. S (J. Serena)., L. R‐T., J. B., J. G‐O., M. P‐O., J. M. F‐R., J. P., M. J., and R. P.; data curation: N. M‐M., J. S (J. Sol)., E. O, M. J., M. P‐O., and R.P.; results discussion, interpretation, and writing—original draft preparation, all authors; writing—review and editing: J. S (J. Sol)., E. O., M. J., and R. P.; visualization: M. J., and R. P.; study supervision: J. M. F‐R., J. P., M. J., and R. P.; funding acquisition: J. M. F‐R., M. J: and R.P. All authors have read and agreed upon the published version of the manuscript. J. M. F‐R., J. P., M. J., and R. P. are the guarantors of this work and, as such, had full access to all the data in the study and takes responsibility for the integrity of the data.

## FUNDING INFORMATION

This research was funded by the Spanish Ministry of Science, Innovation, and Universities (grant RTI2018‐099200‐B‐I00, and PID2022‐143140OB‐I00, co‐funded by the European Regional Development Fund. “A way to build Europe”), and the Generalitat of Catalonia (Agency for Management of University and Research Grants [2021SGR00990] and Department of Health [SLT002/16/00250]) to R.P. The work is also supported by “la Caixa” Foundation (Grant Agreement LCF/PR/HR21/52410002) to M.J. IRBLleida is a CERCA Programme/Generalitat of Catalonia. This work was also partially supported by Instituto de Salud Carlos III (Madrid, Spain) through the research grants PI15/01934, PI18/01022, PI20/01090, and PI21/01361 (co‐funded by the European Regional Development Fund. “A way to make Europe”), and the Catalan Government (AGAUR, #SGR2017‐0734, ICREA Academia Award 2021) to J.M.F‐R.

## CONFLICT OF INTEREST STATEMENT

The authors declare no competing interests.

## INCLUSION AND DIVERSITY

For studies involving human subjects, whether recruited (e.g., clinical analyses) or enrolled spontaneously (e.g., online surveys), we worked to ensure sex balance in the recruitment of human subjects. We worked to ensure ethnic or other types of diversity in the recruitment of human subjects. We worked to ensure that the study questionnaires were prepared in an inclusive way.

## INSTITUTIONAL REVIEW BOARD

The study was conducted according to the guidelines of the Declaration of Helsinki, the guidelines of Spanish legislation (Real Decreto 1716/2011), and the approval of the local ethics committees.

## Supporting information


Dataset S1.

Figures S1–S2.

## Data Availability

The metabolomics dataset used for the analyses is available in https://doi.org/10.34810/data596.
