# Supplementary material for: Plasma acylcarnitines and gut‐derived aromatic amino acids as sex‐specific hub metabolites of the human aging metabolome
Source: Aging Cell. 2023 Mar 23;22(6):e13821. doi: 10.1111/acel.13821 (PMC10265170; doi:10.1111/acel.13821)
Supplement: Supplementary file 2 — Figures S1–S2. [file ACEL-22-e13821-s002.docx]

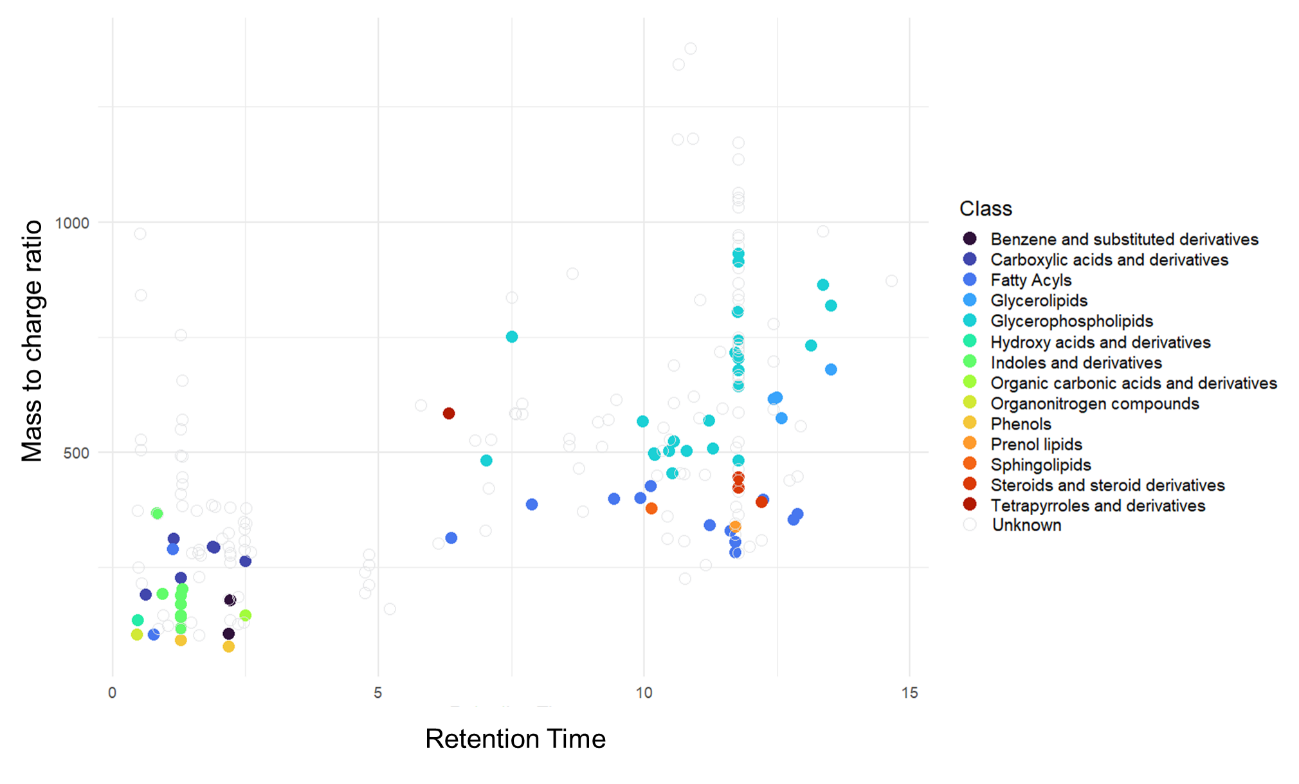


Supplementary Figure 1. Overview of the plasma metabolites significantly associated with age, according to their mass to charge ratio (y axis) and their retention time (x axis). Each metabolic class is represented with a different color. Unidentified molecules are represented as empty dots.


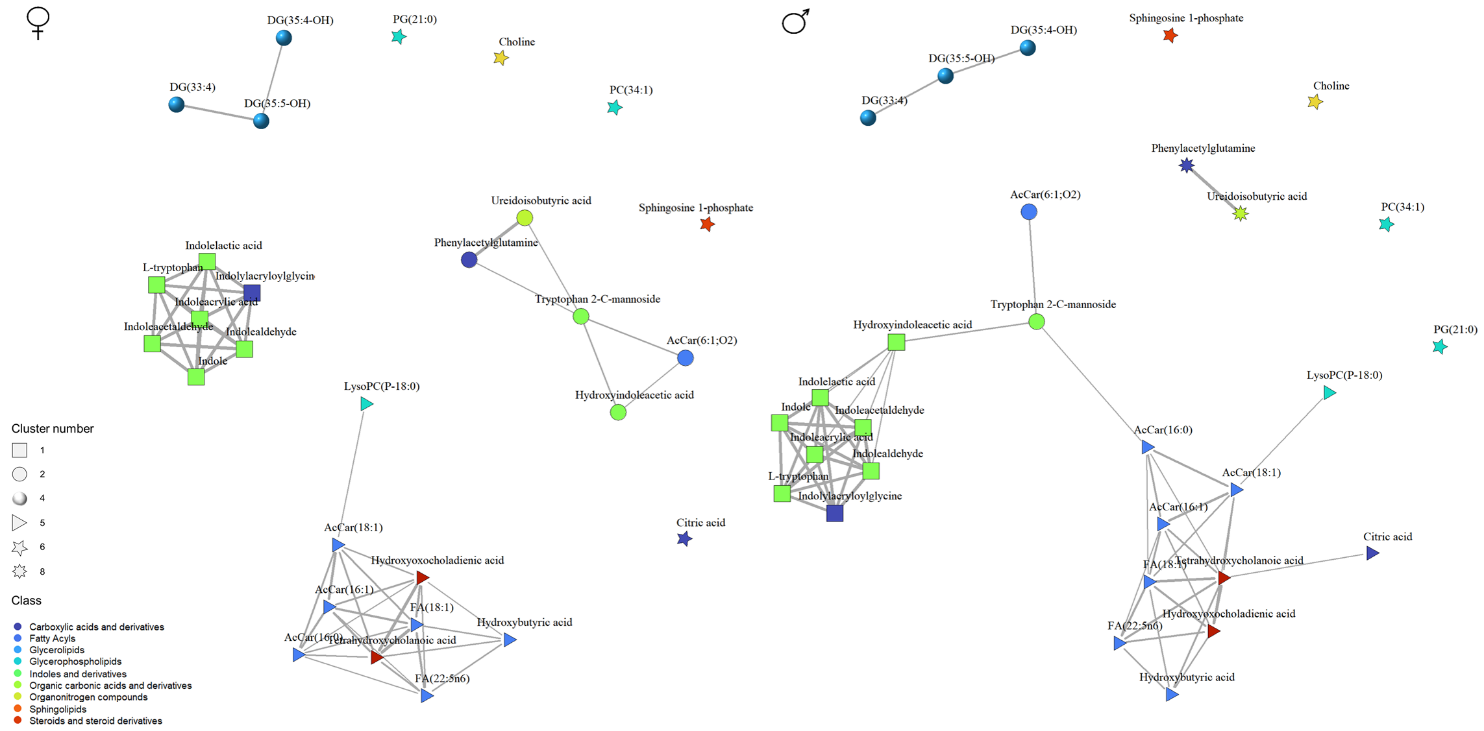


Supplementary Figure 2. Network analysis of the identified plasma metabolites associated with age that were validated in independent cohorts stratifying by gender. Medium and strong correlations (FDR p-value<0.05, Spearman’s rho>0.3) have been used. Each metabolite is represented as a node, colored according to its metabolic class and shaped according to its assigned cluster using a multi-level modularity optimization algorithm. Each correlation is represented as an edge and its width proportional to the correlation coefficient.
